# Emissions from a diesel engine with an air intake system under obstructed conditions

**DOI:** 10.1007/s11356-026-38069-0

**Published:** 2026-07-23

**Authors:** Gabriel de Siqueira Silva, Eduardo Henrique da Silva Santana, Rodney Ferreira Couto, Josué Gomes Delmond, Elton Fialho dos Reis

**Affiliations:** Graduate Program in Agricultural Engineering, State University of Goias, Universidade Estadual de Goiás, Anapolis, Goias Brazil

**Keywords:** Diesel cycle, Airflow, Mechanics, Combustion, Exhaust gases, Pressure drop

## Abstract

Incomplete combustion in internal combustion engines leads to efficiency losses and increases in pollutant emission levels. For combustion to be complete, a well-balanced air–fuel mixture is essential, and this balance depends on the characteristics of the air intake system. In this context, the present study investigated the effect of five restriction levels (0%, 20%, 40%, 60%, and 80%) applied to the air intake duct on diesel engine emissions. To this end, a 75-hp tractor was coupled to an AW Nebraska 800 dynamometer, and gaseous emissions (oxygen, carbon monoxide, carbon dioxide, and nitrogen oxides) were recorded at five engine speeds (1250, 1500, 1750, 2000, and 2250 rpm). The relationship between intake system restriction and combustion quality was assessed, and mild restrictions (up to 20%) promoted more complete combustion under the evaluated operating conditions, as indicated by lower residual oxygen and carbon monoxide concentrations and higher carbon dioxide levels in the exhaust. In contrast, the 60% restriction produced the lowest NOx concentration. Regarding engine speed, higher engine speeds (1500 to 2250 rpm) favored more efficient combustion, with lower oxygen and carbon monoxide levels and higher CO₂ and NOx concentrations. Thus, restricting airflow was found to significantly alter the emissions profile, making it crucial to optimize air intake to balance efficiency and pollutant control.

## Introduction

In diesel engines used in agricultural tractors, which often operate in environments with high dust concentrations, the air intake system becomes particularly prone to obstruction (Dziubak and Karczewski 2022). Incomplete combustion in internal combustion engines is a significant concern, as it leads to the emission of harmful gases and reduces energy efficiency (Seremet and Dinler 2022). Efficient combustion requires an optimized air–fuel mixture, whose quality depends directly on the performance of the intake system.

The intake system of diesel engines consists of the intake pipe, air ducts, air filter, stabilization tank, intake manifold, and resonators, which together must ensure the supply of clean air with minimal restriction, thereby promoting efficient combustion (Lee et al. 2017). In the intake process of naturally aspirated engines, after entering through the duct and passing through the air filter, the air is distributed to the cylinders via the intake manifold, where the pulsating nature of airflow in each cylinder can contribute to increased engine performance within certain speed ranges, while reducing it in others (Reddy and Priyadarsini 2022). Accordingly, the geometry and dimensions of the intake system are designed to meet specific performance objectives, since the cross-sectional area of the ducts directly influences flow velocity and, consequently, the kinetic energy of the admitted air mass, whereas duct length indirectly affects this energy through pressure losses and dynamic effects associated with pressure waves within the system (Costa et al. 2014).

During agricultural operations, one condition that directly affects the performance of agricultural machinery engines is the presence of dust generated by field activities. This dust contains between 60 and 95% silica and alumina. During the air intake process, these particles may reach the lubricating oil, leading to the formation of an abrasive mixture (Dziubak and Boruta 2021). As a result, wear occurs between the internal engine components. Furthermore, Dziubak (2024) and Dziubak and Boruta 2021) reported that silica particles may melt within the cylinder and be transported to the exhaust system, where they form deposits that impair the efficiency of the exhaust gas aftertreatment system.

To mitigate the harmful effects of dust on agricultural tractors, air filtration systems are indispensable, as they serve as a physical barrier that prevents dust particles from reaching internal engine components (Dziubak 2022; Dziubak and Boruta 2021). However, a critical issue arises from the accumulation of particulate matter in the filter elements. The continuous accumulation of dust gradually increases the restriction to airflow through the intake system (Thomas et al. 2013; Dziubak and Boruta 2021).

Heavy-duty machines, such as agricultural tractors, operating under pressure drop conditions ranging from 5 to 12 kPa may experience a loss of maximum engine power. Under extreme conditions, this may lead to air filter failure and compromise the engine combustion system (Dziubak 2022; Dziubak and Boruta 2021). In this context, Li et al. (2023) emphasize that reducing intake airflow resistance is a key measure to increase volumetric efficiency. Consequently, optimizing this process improves combustion performance and reduces pollutant emissions. Recent studies support the close relationship between airflow restriction and increased emissions in diesel engines. Onyegirim et al. (2025) concluded that intake manifold geometry significantly influences engine performance, combustion efficiency, and emission control.

More directly, Dziubak and Karczewski (2022) experimentally demonstrated that increased pressure drop across the air filter significantly alters exhaust gas composition, affecting the concentrations of nitrogen oxides (NOx), carbon monoxide (CO), hydrocarbons (HC), and carbon dioxide (CO₂). Similarly, Demir et al. (2022) observed variations in soot emissions and exhaust gas temperature across different manifold designs, while Yun (2021) optimized an intake system to balance filtration efficiency with minimal flow resistance, a factor that directly influences emissions.

Therefore, understanding the effects of varying degrees of air intake system restriction is essential for both advancing scientific knowledge and practical applications in the agricultural sector. Furthermore, in the Brazilian context, where commercially available diesel fuel is required to contain a biodiesel blend, this knowledge becomes even more relevant. Accordingly, to contribute to this field, the present study aimed to quantify the residual emissions of a diesel engine installed in an agricultural tractor under varying airflow restrictions in the air intake system and at different engine speeds.

## Material and methods

The study was conducted at the Central Campus of the State University of Goiás (UEG), located in the municipality of Anápolis, State of Goiás, Brazil. Geographically, the experimental site is located at 16° 22′ 47″ S, 48° 56′ 53″ W, at an altitude of 1112 m above sea level.

The tractor used in the test had accumulated 1582 operating hours and was equipped with a diesel-cycle engine fueled with S500 diesel. The engine featured four cylinders, a displacement of 3908 cm^3^, and natural aspiration, with a rated power of 55.1 kW (75 hp) at 2500 rpm and a rated torque of 258 N·m at 1500 rpm. It was fitted with a direct injection system comprising a diaphragm feed pump and a rotary injection pump, auxiliary front-wheel drive (FWD), and a power take-off (PTO) with a standard speed of 540 rpm at an engine speed of 1967 rpm, resulting in a transmission ratio of 3.64:1. Table 1 presents the main technical specifications of the engine and fuel used in the experiment.

**Table 1:** Technical specifications of the diesel engine and fuel used in the experiment

Engine	
Model	New Holland S8000
Fuel injection	Direct injection
Displacement	3908 cm^3^
Air filter	Dry
Air–fuel ratio	18:1–70:1
Compression ratio	18.1:1
Rated power	75 hp (55.1 kW) at 2500 rpm
Fuel	
Diesel	S500
Biodiesel content	15%
Maximum sulfur content	500 mg kg^−1^
Kinematic viscosity at 40 °C	2–5 mm^2^ s^−1^
Density at 20 °C	820.3–870.3 kg m^−3^

Table 2 presents the technical specifications of the air filters installed on the tractor before the experiment.

**Table 2:** Technical specifications of the primary and secondary air filters used in the experiment

Characteristic	Primary filter	Second filter
Tecfil code	ARS7109	ASR806
Position	Outer	Inner
Height	≈ 343 mm	343 mm
Outside diameter	≈ 163 mm	77 mm
Inside diameter	≈ 91 mm	62.5 mm
Filter media	Special cellulose	Felt/cellulose

Engine load was controlled using an AW dynamometer, Nebraska 800 model (NEB 800), duly calibrated according to the manufacturer’s recommendations. The unit was fitted with the 2100 DynoPro data acquisition and control system and equipped with a mechanically braked, water-cooled load unit rated at 600 kW (816 hp) at 1000 rpm and 5630 N·m at 1000 rpm. Table 3 summarizes the equipment used in the experiment, along with their measurement ranges and accuracies.

**Table 3:** Equipment used during the experimental tests, their measurement ranges, and accuracies

Equipment	Model	Measured variable	Measurement range	Accuracy
Portable combustion gas analyzer	Testo 340	O₂	0–25 vol.%	± 0.2 vol.%
		CO	0–10,000 ppm	± 10 ppm or ± 10% of reading (0–200 ppm); ± 20 ppm or ± 5% of reading (201–2000 ppm); ± 10% of reading (2001–10,000 ppm)
		CO₂ (calculated)	0–CO₂ max	± 0.2 vol.%
		NO₂	0–500 ppm	± 10 ppm (0–199 ppm); ± 5% of reading (remaining range)
		NO	0–4000 ppm	± 5 ppm (0–99 ppm); ± 5% of reading (100–1999 ppm); ± 10% of reading (2000–4000 ppm)
		Exhaust gas temperature	−40 to 1200 °C	± 0.5 °C (−20 to 99 °C); ± 0.5% of reading (remaining range)
		Differential pressure	−200 to 200 hPa	± 0.5 hPa (−49.9 to 49.9 hPa); ± 1.5% of reading (remaining range)
		Absolute pressure	600–1150 hPa	± 10 hPa
Hydraulic dynamometer	AW Nebraska 800 (NEB 800)	Torque	Up to 5630 N·m	-
		Power	Up to 600 kW	-
Multiparameter weather meter	Akrom KR500	Air temperature	-	-
		Relative humidity	-	-
		Wind speed	-	-

Emissions were measured using a Testo flue gas analyzer, model 340, calibrated by the manufacturer. The instrument enabled measurements of oxygen (O₂) in the range of 0 to 25 vol%, carbon monoxide (CO) in the range of 0 to 10,000 ppm, nitrogen dioxide (NO₂) in the range of 0 to 500 ppm, and nitric oxide (NO) in the range of 0 to 4000 ppm, as well as the calculation of carbon dioxide (CO₂). Nitrogen oxides (NOx) were calculated as the sum of NO and NO₂. The analyzed variables were the concentrations of oxygen (O₂), carbon monoxide (CO), carbon dioxide (CO₂), nitrogen oxides (NOx), nitrogen dioxide (NO₂), and nitric oxide (NO).

Because exhaust gas temperature is influenced by engine speed, Table 4 presents the average exhaust gas temperature measured at each engine speed during the experiment.

**Table 4:** Average exhaust gas temperature at each engine speed during the experiment

Engine speed (rpm)	1250	1500	1750	2000	2250
Exhaust gas temperature (°C)	439.788	550.072	600.679	606.355	592.255

**Fig. 1:**
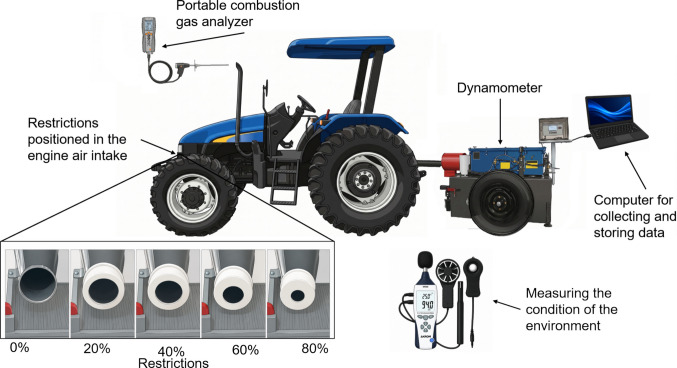
Experimental setup used to evaluate the effects of air intake restriction on engine performance, pressure drop, and exhaust emissions

A schematic representation of the complete experimental setup is shown in Fig. 1. The diagram includes the air intake restriction system, the fuel consumption measurement circuit, the PTO dynamometer setup, the exhaust gas sampling system, and the meteorological monitoring instruments.

To simulate different levels of restriction in the air intake system, the procedure consisted of reducing the free cross-sectional area of the air intake duct at the air filter housing. The tractor used in the experiment was equipped with a dry air filtration system consisting of primary and secondary paper filter elements. The imposed restriction simulated clogged air filters under actual field operating conditions, producing a pressure drop, that varied according to engine speed and obstruction level, as shown in Table 5. The mean pressure drop values ranged from 0.214 to 1.820 kPa, remaining within the acceptable operating limits for naturally aspirated engines, which tolerate maximum intake restrictions ranging from 6.30 to 7.60 kPa.

**Table 5:** Mean pressure drop values ± standard (kPa) deviation in the air intake system as a function of obstruction level and engine speed

Restriction (%)	Engine speed (rpm)				
	2250	2000	1750	1500	1250
0	1.176 ± 0.008	0.975 ± 0.008	0.713 ± 0.011	0.542 ± 0.020	0.377 ± 0.003
20	1.166 ± 0.014	0.908 ± 0.010	0.741 ± 0.019	0.473 ± 0.005	0.343 ± 0.008
40	1.136 ± 0.006	0.983 ± 0.003	0.758 ± 0.007	0.492 ± 0.014	0.214 ± 0.004
60	1.309 ± 0.001	1.144 ± 0.003	0.881 ± 0.006	0.628 ± 0.008	0.432 ± 0.007
80	1.820 ± 0.019	1.639 ± 0.022	1.452 ± 0.026	1.024 ± 0.019	0.686 ± 0.012

The original internal diameter of the air intake duct (0.06504 m) was measured using a digital caliper, allowing the reference cross-sectional area (0.003322 m^2^) to be calculated. The target restriction levels were then systematically defined as reductions of 0% (original unrestricted configuration), 20%, 40%, 60%, and 80% in the reference cross-sectional area.

For each restriction level, the internal diameter required to achieve the target restricted area was calculated. Based on these calculations, cylindrical polyvinyl chloride (PVC) plugs were manufactured with precision to match the internal diameters for each level of area restriction and subsequently fitted into the air intake duct. The resulting geometric parameters for each experimental condition are summarized in Table 6.

**Table 6:** Characteristics of the external duct of the engine air intake system

Restriction (%)		Characteristics of the air intake duct	
	Diameter (m)	Open area (m^2^)	Restricted area (m^2^)
0	0.06504	0.003322	0.000000
20	0.05817	0.002658	0.000664
40	0.05038	0.001993	0.001329
60	0.04113	0.001329	0.001993
80	0.02908	0.000664	0.002658

Before conducting the diesel engine tests, the engine speeds at which data collection would be performed were defined based on several premises. The first premise establishes that the highest operational efficiency during fieldwork is achieved at speeds close to 1400 rpm, which provides maximum torque for tractors (Akal and Selvi 2023). In this regard, an engine speed of 1500 rpm was selected because, according to the manufacturer’s manual of the tractor used in the experiment, maximum torque for this configuration is obtained at this speed.

The second premise assumes that, after the soil preparation phase, tractors are typically operated at approximately 2200 rpm, a speed range close to the maximum power speed (Akal and Selvi 2023). The third premise follows the recommendation of the Organisation for Economic Co-operation and Development (OECD 2024), in its Code 2, which establishes that testing should include an engine speed at least 15% below the maximum torque speed. It is well known that the power take-off (PTO), under standard operating conditions and commonly used in several operations, runs at 540 rpm.

For the tractor used in the test, according to the manufacturer’s manual, this condition is achieved with the engine operating at 1967 rpm. In summary, to encompass both engine speeds typical of field operations and conditions close to maximum torque and power, the following engine speeds were defined for the tests: 1250 rpm (OECD), 1500 rpm (maximum torque), 1750 rpm (operating range), 2000 rpm (PTO), and 2250 rpm (operating range and near maximum power).

It should be noted that, before testing, the tractor underwent maintenance, including replacement of the engine and transmission lubricating oils, as well as replacement of the crankcase oil filter, fuel filter, and the air filter, which consisted of primary and secondary elements. The transmission system was also serviced to ensure the tractor was in proper operating condition, as recommended by the manufacturer. The tractor and dynamometer were positioned on a level surface and coupled to minimize the angle of the driveline shaft connecting the tractor PTO to the dynamometer. Emissions were recorded at the engine exhaust outlet, with a metal bracket fixed at the exhaust exit to keep the gas analyzer probe centered in the exhaust and ensure consistent data collection.

During the tests, meteorological variables were monitored using an Akrom KR500 multiparameter instrument to maintain conditions as close as possible to those recommended by OECD Code 2 (OECD 2024) and to assess the potential influence of environmental conditions on engine performance and exhaust emissions. The experimental measurements were conducted throughout the day, between 08:00 and 16:00 h, resulting in the natural variation of meteorological conditions observed during the testing period. Ambient temperature ranged from 23.1 to 29.6 °C, relative humidity from 14.0 to 29.3%, wind speed from 1.0 to 3.8 m s⁻^1^, and atmospheric pressure from 89.6 to 89.9 kPa.

The variation in atmospheric pressure was minimal (< 0.4%), whereas the observed temperature range resulted in only minor changes in air density. These variations were substantially smaller than the reductions in airflow imposed by the experimental intake restrictions (20–80%) and were therefore considered to have a negligible influence on the overall engine response. Furthermore, exhaust gas measurements were performed using a sampling probe positioned at the center of the exhaust outlet and secured with a metal bracket, thereby minimizing potential interference from external wind conditions during sampling. The environmental conditions recorded during the experimental period are summarized in Table 7.

**Table 7:** Environmental conditions during the experimental period

Variable	Minimum	Maximum
Ambient temperature (°C)	23.1	29.6
Relative humidity (%)	14.0	29.3
Wind speed (m s⁻^1^)	1.0	3.8
Atmospheric pressure (kPa)	89.6	89.9

The experiments were conducted using a completely randomized design (CRD) in a 5 × 5 split-plot factorial arrangement. The main plots corresponded to the five air intake area restriction levels (0%, 20%, 40%, 60%, and 80%), whereas the subplots consisted of the five engine speeds (1250, 1500, 1750, 2000, and 2250 rpm). A total of 25 treatments were evaluated with three replicates each, resulting in 75 experimental units. The analyzed variables were obtained as the average of three measurements.

The mathematical model adopted for the split-plot analysis of variance (ANOVA) is described by the following equation:

$${Y}_{ijk} = \mu +{\tau}_{i}+{\delta}_{ij}+{\beta}_{k}+{\left(\tau \beta \right)}_{ik}+{\epsilon}_{ijk}$$

where

- ${Y}_{ijk}$=observed value of the response variable;
- μ=overall mean;
- ${\tau}_{i}$=effect of the restriction level (main plot treatment);
- ${\delta}_{ij}$=experimental error associated with the main plot;
- ${\beta}_{k}$=effect of engine speed (subplot treatment);
- $\tau \beta$=interaction effect between restriction level and engine speed;
- ${\epsilon}_{ijk}$=experimental error associated with the subplot.

Measurement uncertainty, experimental error, and repeatability were assessed and are reflected in the coefficient of variation (CV) and the residual mean square (MSE) obtained from the analysis of variance.

The data were verified against the basic assumptions for analysis of variance. After verification, the data were evaluated by analysis of variance (ANOVA) and submitted to the *F*-test at a 5% significance level. Because the data were quantitative, the Scott–Knott test was used for mean comparisons, and regression analysis was subsequently performed. R statistical software (R Core Team 2023) was used for the statistical analyses; Microsoft Excel 2016 (Microsoft Corporation 2015) was used to prepare the tables; and Python 3.13.9 (Python Software Foundation 2026) was used to generate the figures using the NumPy and Matplotlib libraries.

## Results and discussion

The concentrations of oxygen (O₂), carbon monoxide (CO), carbon dioxide (CO₂), and nitrogen oxides (NOx) were not significantly influenced by the interaction between the factors (restrictions and engine speeds), according to the analysis of variance (Table 8). However, these variables were significantly affected by each factor individually.

**Table 8:** Analysis of variance (ANOVA) for oxygen (O₂), carbon monoxide (CO), carbon dioxide (CO₂), and nitrogen oxides (NOx) concentrations in the exhaust gases

Sources of variation	Degrees of freedom	Mean square			
		O_2_	CO	CO_2_	NOx
Restrictions (1)	4	10.93*	1.557811 × 10^6^**	6.04*	98,940.44**
Residual	10	2.16	2.044174 × 10^5^	1.19	7646.77
Engine speeds (2)	4	47.07**	1.339329 × 10^7^**	28.51**	55,338.68**
1 × 2	16	0.84	1.263433 × 10^5^	0.47	7454.90
Residual	40	0.79	6.674076 × 10^4^	0.45	4356.36
Total	74	-	-	-	-
Means	-	8.79	1682.81	8.95	772.50
CV 1 (%)	-	16.70	26.87	12.20	11.32
CV 2 (%)	-	10.12	15.35	7.49	8.54

*CV*, coefficient of variation; *significant at the 5% probability level by the *F*-test; **significant at the 1% probability level by the *F*-test

**Fig. 2:**
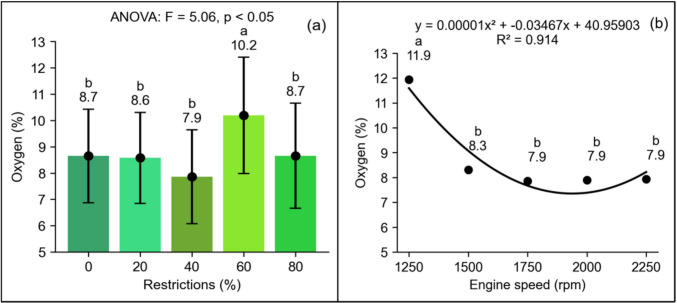
Oxygen concentration (%, *y*-axis) as a function of **a** different air intake system restriction levels (%, *x*-axis) and **b** different engine speeds (rpm, *x*-axis). Vertical bars in graph **a** represent the standard deviation. Experimental conditions: measurements were performed under load using a dynamometer

The residual oxygen content (%) in the exhaust gases under the different air intake system restriction levels is shown in Fig. 2a. On average, the highest residual oxygen value in the exhaust was recorded at the 60% restriction (10.2% ± 2.21%), whereas the 0% (8.7% ± 1.78%), 20% (8.6% ± 1.73%), 40% (7.9% ± 1.78%), and 80% (8.7% ± 2.0%) restrictions did not differ from one another and represented the lowest mean values. Although the mean-comparison test detected statistical differences, the oxygen values were close, ranging from 7.9% to 10.2%.

When the lower intake restriction levels (0%, 20%, and 40%) were considered, these treatments yielded the lowest residual oxygen concentrations in the exhaust, indicating more efficient combustion and greater oxygen consumption. From the perspective of combustion chemistry, greater oxygen consumption in the combustion chamber leads to more complete fuel combustion, thereby reducing products of incomplete combustion (Ghany et al. 2024). The values obtained in the present study fall within the range of 3% to 17% reported in the literature for diesel engine exhaust gases under different load conditions (Kopseak et al. 2022).

The relationship between oxygen concentration (%) and engine speed is presented in Fig. 2b. The second-order polynomial regression model indicated a decrease in oxygen concentration between 1250 and 1750 rpm. The highest residual oxygen concentration (11.9%) was observed at 1250 rpm and can be attributed to lower combustion efficiency under this operating condition. At engine speeds ranging from 1500 to 2250 rpm, the residual oxygen concentration stabilized at 7.9%–8.3%, with no statistically significant differences among these speeds. This behavior may have occurred because, at low engine speeds and under the load imposed by the dynamometer, cylinder filling is limited, thereby reducing the mass of intake air. As a result, combustion becomes less complete, leading to a higher residual oxygen concentration in the exhaust gases (Kose and Acaroglu 2020). This behavior is consistent with the findings of Sethin et al. (2024), who reported a decrease in exhaust oxygen concentration with increasing engine speed. According to these authors, higher engine speeds are generally associated with greater airflow and improved combustion efficiency.

**Fig. 3:**
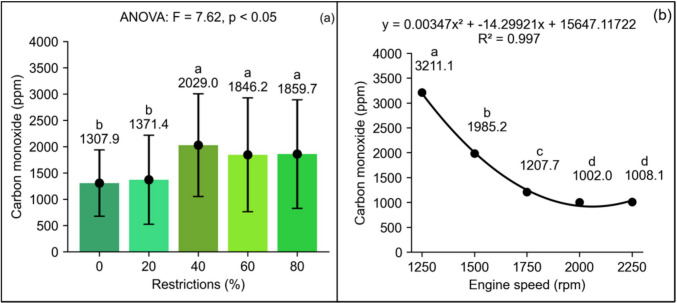
Carbon monoxide concentration (ppm, *y*-axis) as a function of **a** different air intake system restriction levels (%, *x*-axis) and **b** different engine speeds (rpm, *x*-axis). Vertical bars in graph **a** represent the standard deviation. Experimental conditions: measurements were performed under load using a dynamometer

For carbon monoxide (CO), as shown in Fig. 3a, the highest emissions were observed at the 40%, 60%, and 80% restriction levels, corresponding to the conditions in which the air intake flow area was reduced. As explained by Ahmadipour et al. (2019), one of the most important factors affecting CO emissions is the air–fuel ratio, as incomplete or inefficient combustion indicates insufficient air in the mixture. Another fundamental aspect of combustion chemistry is that oxygen deficiency under higher intake restrictions promotes incomplete combustion, markedly increasing the formation of unburned hydrocarbons (HC). Like CO, HC emissions are direct products of locally fuel-rich zones that lack sufficient oxygen for complete oxidation to CO₂.

Across the different restriction levels, CO concentrations ranged from 1307.9 to 2029 ppm, with the 0% and 20% restriction levels producing the lowest mean emissions (1307.9 and 1371.4 ppm, respectively). When these values are compared with those reported in other studies, differences are observed; however, these differences may be attributed to variations in engine rated power, fuel characteristics, and the air–fuel mixture. According to Amsal et al. (2023), leaner mixtures (excess air) produce lower CO concentrations because CO itself plays a fundamental role in the oxidation of hydrocarbons.

Fuel-rich mixtures produce higher CO (and HC) concentrations because of the excess unoxidized fuel. Iacono et al. (2024), while evaluating the emissions and performance of a 75.8 kW agricultural tractor, reported CO emissions of up to approximately 2678 ppm. Emaish et al. (2021) evaluated a 66.2 kW diesel engine and reported CO emissions of approximately 369 ppm. Dziubak et al. (2023), in a study investigating the effect of the air filter on a 338 kW diesel engine, reported CO emissions of approximately 800 ppm.

The relationship between carbon monoxide concentration and engine speed was described by a quadratic regression model, which indicated a decrease in CO emissions from 1250 to 2000 rpm (Fig. 3b). CO emissions ranged from 3211.1 ppm at 1250 rpm to 1002 ppm at 2000 rpm. At low engine speeds and under certain load conditions, reduced airflow turbulence and poorer air–fuel mixture formation may result in incomplete combustion, thereby promoting the presence of unburned fuel (Karami et al. 2020).

Incomplete combustion leads to higher carbon monoxide emissions, primarily because of poor air–fuel mixing or oxygen deficiency during combustion under load (Karthikeyan et al. 2021). Consistent with this behavior, Kariuki et al. (2024), while investigating the emissions of a 3.5 kW diesel engine, observed that CO emissions decreased with increasing engine load and reported a maximum CO concentration of 375 ppm. According to the authors, this pattern occurs because increasing engine load requires a greater fuel supply; when the air–fuel mixture becomes fuel-rich (excess fuel), combustion is incomplete, resulting in higher CO and HC emissions.

**Fig. 4:**
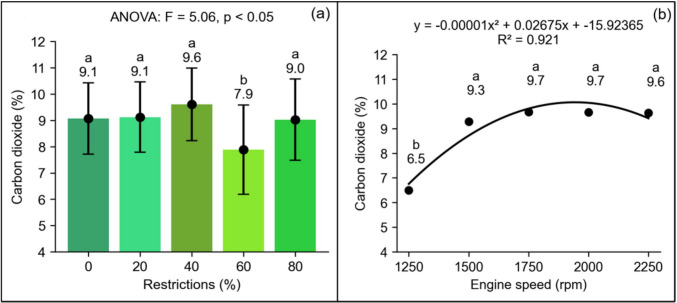
Carbon dioxide concentration (%, *y*-axis) as a function of **a** different air intake system restriction levels (%, *x*-axis) and **b** different engine speeds (rpm, *x*-axis). Vertical bars in graph **a** represent the standard deviation. Experimental conditions: measurements were performed under load using a dynamometer

Air intake system restrictions significantly affected carbon dioxide (CO₂) concentrations. The mean comparison test revealed significant differences among the restriction levels evaluated. The 0%, 20%, 40%, and 80% restriction levels (9.1%, 9.1%, 9.6%, and 9.0%, respectively) produced the highest mean CO₂ concentrations (group “a”). In contrast, the 60% restriction level (7.9%) resulted in the lowest mean CO₂ concentration and differed statistically from all other restriction levels (Fig. 4a).

CO₂ concentrations varied only slightly, ranging from 7.9% to 9.6%. The higher CO₂ concentrations observed at the lower restriction levels can be explained by the findings of Jeevahan et al. (2017), who reported that CO₂ formation results from the complete oxidation of fuel. Under conditions of adequate oxygen availability, the conversion of CO to CO₂ is favored, whereas oxygen deficiency impairs this process. Ghotto et al. (2025) reported that carbon monoxide is formed when the carbon in the fuel is not completely oxidized to CO₂, primarily because of insufficient oxygen, lower combustion temperatures, or inefficient fuel–air mixing.

The relationship between engine speed and carbon dioxide (CO₂) concentration exhibited a significant pattern, which was described by a quadratic regression model (Fig. 4b). An increase in CO₂ concentration was observed with the initial increase in engine speed. At 1250 rpm, the CO₂ concentration was 6.5%. Increasing the engine speed to 1500 rpm resulted in a marked increase in CO₂ concentration to 9.3%. Thereafter, CO₂ concentration stabilized, reaching 9.7% at 1750 rpm, 9.7% at 2000 rpm, and 9.6% at 2250 rpm. The assignment of the same statistical group (“a”) to all engine speeds between 1500 and 2250 rpm confirms the absence of significant differences in CO₂ concentration within this range, indicating stabilization. This behavior is consistent with the findings of Kose and Acaroglu (2020), who reported that combustion is less efficient at low engine speeds, resulting in lower CO₂ concentrations. As engine speed increases, the greater airflow improves combustion efficiency, thereby increasing CO₂ production.

An inverse relationship was also observed between residual oxygen and carbon dioxide concentrations. At the engine speed that produced the highest oxygen concentration in the exhaust, the CO₂ concentration was the lowest, and vice versa. This behavior confirms the direct relationship between combustion efficiency, oxygen consumption, and CO₂ formation. These results suggest that, from this engine speed range onward, the combustion process becomes more complete, thereby promoting the oxidation of CO to CO₂. As reported by Bhangwar et al. (2024), carbon dioxide is a primary by-product of complete combustion, supporting the interpretation that combustion efficiency is optimized at moderate engine speeds.

**Fig. 5:**
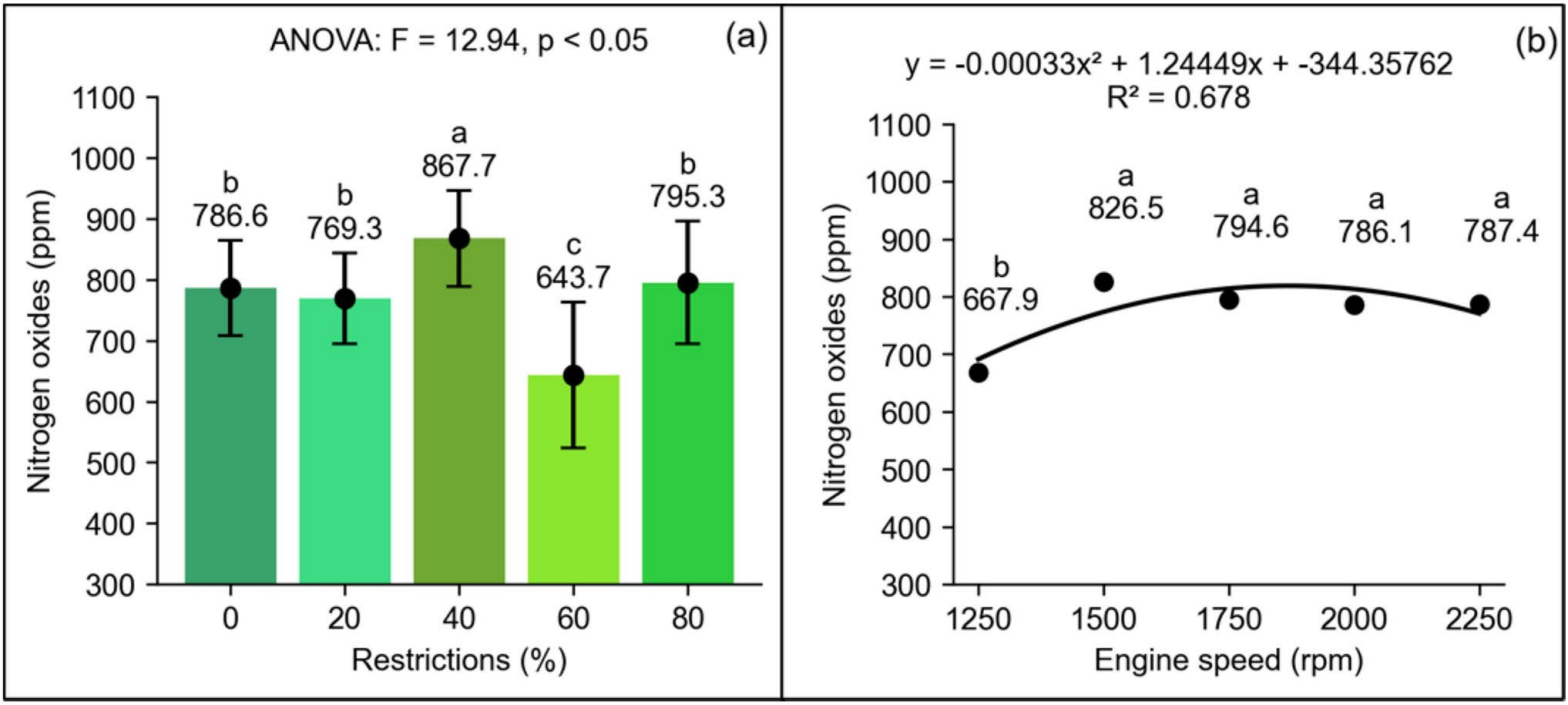
Nitrogen oxides (NOx) concentration (ppm, *y*-axis) as a function of **a** different air intake system restriction levels (%, *x*-axis) and **b** different engine speeds (rpm, *x*-axis). Vertical bars in graph **a** represent the standard deviation. Experimental conditions: measurements were performed under load using a dynamometer

The highest nitrogen oxides (NOx) concentration was observed at the 40% air intake system restriction level (867.7 ppm). In contrast, the lowest concentration was recorded at the 60% restriction level (643.7 ppm). Conversely, the remaining restriction levels (0%, 20%, and 80%) exhibited intermediate mean concentrations that did not differ statistically from one another (786.6, 769.3, and 795.3 ppm, respectively) (Fig. 5a).

NOx formation during engine combustion is influenced by the characteristics of the combustion chamber (Wang and Yang 2024), as the combustion reactions occur in a high-temperature environment (approximately 2800 K) and are further affected by the turbulent flow within the chamber. It is well established that high combustion temperatures and high O₂ concentrations favor NOx formation. Higher flame temperatures are generally associated with increased NOx formation (Radhouane et al. 2023). Nitrogen oxides are generated in high-temperature zones, and their occurrence depends on the heat release rate (Wright and Lewis 2022). The reduction in NOx concentration observed at the 60% restriction level can be explained by the severe reduction in intake air mass due to the high degree of restriction, which lowers combustion temperature and combustion efficiency, thereby directly inhibiting NOx formation.

The relationship between NOx concentration and engine speed (Fig. 5b) was best described by a quadratic regression model, which indicated a peak in NOx concentration between 1500 and 1750 rpm. The lowest NOx concentration was observed at 1250 rpm (667.9 ppm). However, higher concentrations were recorded at 1500, 1750, 2000, and 2250 rpm (826.5, 794.6, 786.1, and 787.4 ppm, respectively), with no statistically significant differences among these engine speeds. The reduction in NOx concentration observed as engine speed decreased from 1500 to 1250 rpm under the high-load, low-speed operating conditions typical of dynamometer testing can be attributed to locally limited oxygen availability, lower peak combustion temperatures, and a greater predominance of diffusion-controlled combustion. These factors inhibit the thermal NOx formation mechanisms, whose kinetics are strongly dependent on temperature and the residence time of gases in high-temperature regions (Heywood 2018; Turns 2012).

In addition to the gases discussed above, the influence of combustion on sulfur oxides (SOx) emissions should also be considered. Because the engine was fueled with S500 diesel fuel, SOx emissions are a direct consequence of the fuel sulfur content (500 ppm). Their formation is proportional to fuel consumption and the dynamometer load, directly reflecting the physicochemical characteristics of the commercial diesel fuel used.

By comparing the engine speed ranges and emission profiles, it can be concluded that operating the tractor at 1500–2000 rpm, combined with low air filter restriction levels (0–40%), represents the optimum operating condition. This operating range ensures maximum torque at 1500 rpm, stabilizes fuel consumption, maximizes CO₂ formation (an indicator of complete combustion), and mitigates peak emissions of incomplete combustion products, such as CO and HC, such as CO and HC.

## Conclusions

1. At the 0%, 20%, and 40% restriction levels, lower residual O₂ concentrations were observed, consistent with greater oxygen consumption during combustion.
2. The 0% and 20% restriction levels resulted in the lowest CO concentrations, indicating a higher degree of complete combustion.
3. The 0%, 20%, and 40% restriction levels produced the highest CO₂ concentrations, consistent with greater carbon conversion during combustion.
4. The 60% restriction level resulted in the lowest NOx concentrations because of lower peak flame temperatures and reduced oxygen availability.
5. Engine speeds between 1500 and 2250 rpm resulted in lower residual O₂ concentrations in the exhaust, consistent with more complete combustion.
6. The lowest CO concentrations were observed at 2000 and 2250 rpm, indicating a higher degree of combustion completeness at these engine speeds.
7. Engine speeds between 1500 and 2250 rpm produced the highest CO₂ concentrations, consistent with more complete combustion within this operating range.
8. The highest NOx concentrations were observed between 1500 and 2250 rpm, suggesting combustion temperatures favorable to NOx formation.
9. Oxygen deficiency under higher intake restriction levels also increases the formation of unburned hydrocarbons (HC), following the same trend observed for CO.
10. SOx emissions are inherent to the characteristics of the S500 diesel fuel used and are directly proportional to engine load.
11. Preventive maintenance of the air filter is essential, and air intake restriction should not exceed 40% to avoid power loss and increased pollutant emissions.
12. The optimum operating range is between 1500 and 2000 rpm, ensuring maximum torque, improved combustion efficiency, and mitigation of pollutant emissions.

## Data Availability

All data generated from this study are presented in tables and figures; the raw data will be available upon reasonable request to the corresponding author.
